# A network embedding-based multiple information integration method for the MiRNA-disease association prediction

**DOI:** 10.1186/s12859-019-3063-3

**Published:** 2019-09-12

**Authors:** Yuchong Gong, Yanqing Niu, Wen Zhang, Xiaohong Li

**Affiliations:** 1 School of Computer Science, Wuhan University, Wuhan, 430072 China; 2 School of Mathematics and Statistics, South-Central University for Nationalities, Wuhan, 430074 China; 3 College of Informatics, Huazhong Agricultural University, Wuhan, 430070 China

**Keywords:** miRNA-disease associations, Network embedding, Random forest

## Abstract

**Background:**

MiRNAs play significant roles in many fundamental and important biological processes, and predicting potential miRNA-disease associations makes contributions to understanding the molecular mechanism of human diseases. Existing state-of-the-art methods make use of miRNA-target associations, miRNA-family associations, miRNA functional similarity, disease semantic similarity and known miRNA-disease associations, but the known miRNA-disease associations are not well exploited.

**Results:**

In this paper, a network embedding-based multiple information integration method (NEMII) is proposed for the miRNA-disease association prediction. First, known miRNA-disease associations are formulated as a bipartite network, and the network embedding method Structural Deep Network Embedding (SDNE) is adopted to learn embeddings of nodes in the bipartite network. Second, the embedding representations of miRNAs and diseases are combined with biological features about miRNAs and diseases (miRNA-family associations and disease semantic similarities) to represent miRNA-disease pairs. Third, the prediction models are constructed based on the miRNA-disease pairs by using the random forest. In computational experiments, NEMII achieves high-accuracy performances and outperforms other state-of-the-art methods: GRNMF, NTSMDA and PBMDA. The usefulness of NEMII is further validated by case studies. The studies demonstrate the great potential of network embedding method for the miRNA-disease association prediction, and SDNE outperforms other popular network embedding methods: DeepWalk, High-Order Proximity preserved Embedding (HOPE) and Laplacian Eigenmaps (LE).

**Conclusion:**

We propose a new method, named NEMII, for predicting miRNA-disease associations, which has great potential to benefit the field of miRNA-disease association prediction.

**Electronic supplementary material:**

The online version of this article (10.1186/s12859-019-3063-3) contains supplementary material, which is available to authorized users.

## Background

MiRNAs are a kind of small non-coding RNA molecules containing about 22 nucleotides, which are involved in the regulation of post-transcriptional gene expression in plants and animals [1]. MiRNAs are usually considered as negative gene regulators, which regulate the expression of messenger RNAs in a sequence-specific manner and repress the protein translation of their target genes. However, studies showed that miRNAs also act as positive regulators. For example, two well-studied miRNAs: Let-7 and the synthetic miRNA miRcxcr4 induce translation upregulation of target messenger RNAs on cell cycle arrest [2]. The increasing evidence demonstrated that miRNAs play critical roles in important biological processes, such as cell growth [3], tissue differentiation [4], cell proliferation [5], embryonic development and apoptosis [6, 7]. More importantly, plenty of miRNAs have been discovered to be related to a wide range of human diseases, such as breast cancer, heart diseases and cardiovascular disease [8–10]. Therefore, the identification of miRNA-disease associations is significant for understanding the molecular mechanisms of human diseases and promoting the diagnosis and treatment of human diseases. Experimental determination of miRNA-disease associations is tremendously expensive and laborious, and has a high failure rate. Therefore, identifying miRNA-disease associations through computational approaches attracts wide attention from scientific communities.

In the past few years, plenty of computational methods have been developed to predict miRNA-disease associations. For example, Sun et al. [11] proposed a method named NTSMDA, which used the network topological information and the network-based resource allocation algorithm to predict miRNA-disease associations. You et al. [12] constructed a heterogeneous graph by integrating miRNA-disease associations, miRNA-miRNA similarities and disease-disease similarities, and developed a network path-based computational method. Chen et al. [13] proposed a method called RKNNMDA, which implemented k-nearest-neighbor algorithm to select candidate miRNAs (or diseases) and used ranking support vector machine (SVM) to rank candidates and make predictions. Xiao et al. [14] proposed a graph regularized non-negative matrix factorization method called GRNMF, which integrated the disease semantic information, miRNA functional information and miRNA-disease associations. Chen et al. [15] proposed an inductive matrix completion method named IMCMDA by integrating miRNA functional similarity, disease semantic similarity and Gaussian interaction profile kernel similarity. Luo et al. [16] proposed a novel semi-supervised prediction method named MDAGRF based on the graph regularization framework. Chen et al. [17] proposed a bipartite network projection-based method named BNPMDA based on the rating-integrated bipartite network recommendation and the known miRNA-disease associations.

Existing state-of-the-art methods make use of miRNA-target associations, miRNA-family associations, miRNA functional similarity, disease semantic similarity and known miRNA-disease associations. However, the known miRNA-disease associations are not well exploited. To the best of our knowledge, known miRNA-disease associations can be formed as a bipartite network, but features from the network are seldom considered. The network embedding is to learn embedding representations of nodes by preserving the property of the network. Recently, the network embedding methods, such as DeepWalk [18] and node2vec [19], have been applied to many bioinformatics problems and produced good performances. For example, Zong et al. [20] utilized node embeddings learned by DeepWalk in a heterogeneous network to calculate drug-drug similarity and target-target similarity, and then predicted novel drug-target associations. Li et al. [21] proposed a similarity-based miRNA-disease prediction method, which used DeepWalk to obtain node embeddings and then calculated cosine similarities. Liu et al. [22] used node2vec to obtain node embeddings, and then utilized them to train random forest model for protein complexes identification.

In this paper, a network embedding-based multiple information integration method (NEMII) is proposed for the miRNA-disease association prediction. First, known miRNA-disease associations are formulated as a bipartite network, and the network embedding method Structural Deep Network Embedding (SDNE) is adopted to learn node embeddings in the bipartite network. Second, the embedding representations of miRNAs and diseases are combined with biological features about miRNAs and diseases to represent miRNA-disease pairs. Third, prediction models are constructed based on the miRNA-disease pairs by using random forest. In computational experiments, NEMII achieves high-accuracy performances and outperforms other state-of-the-art methods: GRNMF, NTSMDA and PBMDA. The usefulness of NEMII is further validated by case studies. The studies demonstrate the great potential of network embedding methods for the miRNA-disease association prediction, and the embedding method SDNE outperforms other popular network embedding methods: DeepWalk, High-Order Proximity preserved Embedding (HOPE) and Laplacian Eigenmaps (LE).

## Results

### Evaluation metrics

In a miRNA-disease bipartite network, non-association miRNA-disease pairs are much more than association pairs. The miRNA-disease association prediction is a semi-supervised learning task, and the key point is to predict undiscovered miRNA-disease associations from all non-association miRNA-disease pairs. We adopt five-fold cross-validation to evaluate the performances of prediction models. Additional file 1: Figure S1 shows how to implement five-fold cross-validation. The known miRNA-disease associations are randomly equally divided into five subsets. In each fold, one subset of associations is removed, and we can train a prediction model only based on the remaining four subsets of associations. In the stage of training, SDNE is to learn embeddings of miRNAs and diseases from the network with remaining four subsets of associations. Then, the embeddings are combined with biological features about miRNAs and diseases to represent miRNA-disease pairs. Four subsets of associations (miRNA-disease pairs) are naturally used as positive instances. For the semi-supervised learning task, all other pairs (non-association) can be used as negative instances. Therefore, a RF-based prediction model is constructed. In the stage of prediction, the prediction model makes prediction for all non-association miRNA-disease pairs, which include the removed associations and real non-association pairs. Then, the prediction scores and their real labels about these pairs are used to calculate the metric scores. To avoid the bias of data split, we implement 10 runs of five-fold cross-validation for each model, and average performance is adopted.

We adopt several evaluation metrics: the area under the precise-recall curve (AUPR) and the area under the receiver-operating characteristic curve (AUC), F1-measure (F1), recall (REC) and precision (PRE), accuracy (ACC) and specificity (SPEC).

### Discussion of NEMII

NEMII combines three types of feature vectors for describing miRNA-disease pairs, i.e. vectors based on miRNA-family associations, vectors based on disease semantic similarity and vectors based on Structural Deep Network Embedding (SDNE). Vectors based on Structural Deep Network Embedding (SDNE) are obtained from the known miRNA-disease bipartite network.

First, we try to discuss the influence of different feature combinations on the performance of NEMII. We consider different combinations of these three features: miRNA-family, disease similarity and SDNE feature by combining their feature vectors, and build the corresponding random forest-based prediction models. All models based on feature combinations are evaluated by 10 runs of five-fold cross-validation, and the results are shown in Table 1. In general, combinations with SDNE feature produce better performance than combinations without SDNE feature. For example, the AUPR score of combination 2 (without SDNE feature) is around 57% lower than that of the other four combinations (with SDNE feature). The AUC score of combination 2 (without SDNE feature) is 14% lower than that of other four combinations (with SDNE feature). Therefore, the results suggest that SDNE feature plays an important role in the prediction of miRNA-disease associations. Moreover, NEMII models which make use of SDNE feature, miRNA-family feature and disease similarity feature (combination 5) performs better than the models based on other combinations, indicating that the proposed method can well combine diverse features to achieve high-accuracy performances. To further demonstrate the advantage of NEMII, we conduct the statistical analysis to test the difference between NEMII and other feature combinations in terms of AUPR scores. The results show that although there is no significant difference between NEMII and the model based on combination 3 (*p*-value = 0.3449 by one-way ANOVA followed by post hoc Tukey test), and the model based on combination 4 (*p*-value = 0.2759), the NEMII model produces significantly better results than the model based on combination 1 (*p*-value = 0.001) and the model based on combination 2 (*p*-value = 0.001). The results demonstrate that NEMII which integrates SDNE feature, miRNA-family feature and disease similarity feature can produce good performance in the prediction of miRNA-disease associations.

**Table 1:** Performance of NEMII based on different feature combinations

	AUPR	AUC	F1	ACC	REC	SPEC	PRE
combination 1	0.6036 ± 0.0018	0.9252 ± 0.0014	0.6072 ± 0.0020	0.9955 ± 0.0001	0.4860 ± 0.0052	0.9992 ± 0.0001	0.8128 ± 0.0158
combination 2	0.2630 ± 0.0032	0.7890 ± 0.0056	0.3338 ± 0.0032	0.9933 ± 0.0000	0.2360 ± 0.0025	0.9987 ± 0.0000	0.5681 ± 0.0058
combination 3	0.6086 ± 0.0015	0.9284 ± 0.0012	0.6129 ± 0.0031	0.9956 ± 0.0001	0.4887 ± 0.0069	0.9992 ± 0.0001	0.8247 ± 0.0160
combination 4	0.6085 ± 0.0024	0.9262 ± 0.0018	0.6115 ± 0.0026	0.9956 ± 0.0000	0.4836 ± 0.0055	0.9993 ± 0.0001	0.8366 ± 0.0105
combination 5	0.6104 ± 0.0012	0.9293 ± 0.0017	0.6147 ± 0.0025	0.9956 ± 0.0001	0.4893 ± 0.0060	0.9993 ± 0.0001	0.8289 ± 0.0164

^*^ combination 1: SDNE feature alone

^*^ combination 2: miRNA-family feature and disease similarity feature

^*^ combination 3: SDNE feature and miRNA-family feature

^*^ combination 4: SDNE feature and disease similarity feature

^*^ combination 5: SDNE feature, miRNA-family feature and disease similarity feature

The known miRNA-disease associations are important factors for predicting unobserved miRNA-disease associations. In order to test the influence of the number of known associations, i.e. data richness, we randomly remove 10, 20, 30% known miRNA-disease associations from our dataset respectively, and then we perform 10 runs of five-fold cross-validation to evaluate NEMII on the datasets with fewer associations. As shown in Table 2, data richness greatly influenced the performance of our model, and AUPR and AUC scores decrease as more associations are removed. For example, the AUPR score is 0.6104 when there are no associations removed, but it decreases to 0.6001 when removing 10% associations. Then, the AUPR score decreases from 0.5956 (20% associations removed) to 0.5863 (30% associations removed). The AUC score also decreases as associations are removed. More specifically, 10% decrease of associations can lead to around 0.1% decrease of the AUC score. Although the performances of NEMII decrease when reducing associations, NEMII still produces satisfying and robust results in the miRNA-disease predictions. The results demonstrate that SDNE is a robust embedding learning method, and can perform well even if the network becomes sparser.

**Table 2:** Performances of NEMII on datasets with fewer associations

Ratio	AUPR	AUC	F1	ACC	REC	SPEC	PRE
0%	0.6104 ± 0.0012	0.9293 ± 0.0017	0.6147 ± 0.0025	0.9956 ± 0.0001	0.4893 ± 0.0060	0.9993 ± 0.0001	0.8289 ± 0.0164
10%	0.6001 ± 0.0018	0.9276 ± 0.0011	0.6045 ± 0.0037	0.9969 ± 0.0001	0.4811 ± 0.0051	0.9993 ± 0.0001	0.8176 ± 0.0168
20%	0.5956 ± 0.0030	0.9266 ± 0.0014	0.6036 ± 0.0040	0.9965 ± 0.0000	0.4738 ± 0.0091	0.9995 ± 0.0001	0.8354 ± 0.0169
30%	0.5863 ± 0.0026	0.9255 ± 0.0010	0.5946 ± 0.0036	0.9960 ± 0.0001	0.4620 ± 0.0074	0.9995 ± 0.0001	0.8390 ± 0.0290

### Comparison with other network embeddings and other classifiers

As discussed in Discussion of NEMII Section, features extracted by the embedding method SDNE are critical for building NEMII models. To demonstrate the advantage of SDNE, we also consider other popular network embedding methods: Laplacian Eigenmaps (LE) [23], High-Order Proximity preserved Embedding (HOPE) [24] and DeepWalk [18], and compare them with SDNE. LE keeps embeddings of two nodes close when these two nodes have high similarity. HOPE preserves high order proximity by decomposing the similarity matrix and using a generalized Singular Value Decomposition (SVD). DeepWalk uses random walks on graphs to learn latent representations of nodes and encodes them in a continuous space. These embedding methods usually have different parameters, and we set their parameters according to their publication and mainly discuss a common parameter: the dimension of node embeddings.

**Fig. 1:**
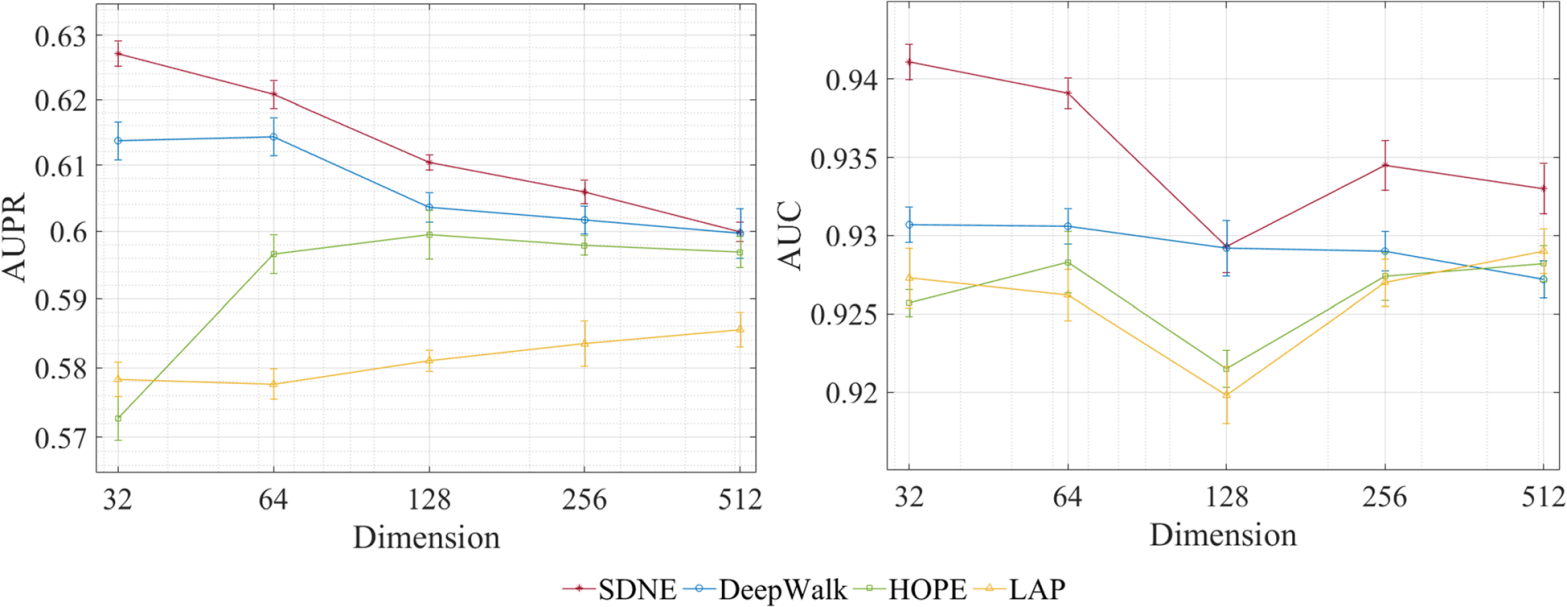
AUPR and AUC of embedding methods based on different dimensions

Here, we discuss the model performance under the different dimensions of node embeddings, ranging from 32 to 512 (2^*k*^, *k* = 5, 6, 7, 8, 9). We respectively adopt these embedding methods to extract embedding features from the network, and then combine them with miRNA-family feature and disease similarity feature to build similar SDNE models. The results of all models are shown in Fig. 1. The y-axis denotes the AUPR and AUC scores obtained by the corresponding model, and the x-axis denotes different dimensions of node embeddings. Clearly, the model using SDNE embedding leads to better AUPR scores and AUC scores than the models using other three embeddings over the different dimensions. To further demonstrate the advantage of SDNE, we conduct one-way ANOVA followed by post hoc Tukey test to test the difference between SDNE and other embedding methods in terms of AUPR scores. The results show that the SDNE model produces significantly better results than the HOPE model (*p*-value = 0.0027) and the LAP model (*p*-value = 0.0019). The *p*-value between the SDNE model and the DeepWalk model is 0.0507, which suggests that there is no significant difference between the SDNE model and the DeepWalk model. Therefore, we conclude that SDNE method can learn more effective node embeddings in the miRNA-disease bipartite network and performs better than other three methods, because the miRNA-disease bipartite network is sparse and SDNE method was proved to be robust to sparse networks [25]. Anothers reason why SDNE works better than other embedding learning methods is that SDNE combines the autoencoder objective with the Laplacian eigenmaps objective.

Moreover, we observe from Fig. 1 (right) that the SDNE model using 128 dimensions produces the lowest AUC score of 0.9293. In order to avoid overestimating our model, we set the embedding vectors of SDNE as 128 dimensions in this study.

In order to show the advantage of random forest (RF) classifier, naive Bayes (NB), logistic regression (LR) and support vector machine (SVM) are used for comparison. By considering the number of trees ranging from 10 to 500 in a step of 10, we set the number of trees in a RF classifier as 350 according to the performances of corresponding models. We adopt the radial basis function for SVM, and use the grid search to obtain optimal parameters C = 1.0 and gamma = 0.1. The prediction models based on different classifiers are evaluated by five-fold cross-validation, and the results are shown in Table 3. Clearly, the model using RF classifier (AUC:0.9293, AUPR: 0.6104) performs better than the models using NB (AUC: 0.9103, AUPR: 0.1846), LR (AUC: 0.9023, AUPR: 0.2129) and SVM (AUC: 0.9021, AUPR: 0.0968). The results demonstrate that RF classifier is suitable for the miRNA-disease association prediction, because RF classifier is effective for the imbalanced and high-dimensional datasets.

**Table 3:** Performance of models based on different classifiers

Classifiers	AUPR	AUC	F1	ACC	REC	SPEC	PRE
RF	0.6104 ± 0.0012	0.9293 ± 0.0017	0.6147 ± 0.0025	0.9956 ± 0.0001	0.4893 ± 0.006	0.9993 ± 0.0001	0.8289 ± 0.0164
NB	0.1846 ± 0.0008	0.9103 ± 0.0089	0.2528 ± 0.0028	0.9892 ± 0.0004	0.2572 ± 0.0124	0.9944 ± 0.0005	0.2532 ± 0.0056
LR	0.2129 ± 0.0008	0.9023 ± 0.0008	0.2734 ± 0.0017	0.9884 ± 0.0004	0.3078 ± 0.0094	0.9933 ± 0.0005	0.2480 ± 0.0096
SVM	0.0968 ± 0.0034	0.9021 ± 0.0010	0.1718 ± 0.0036	0.9740 ± 0.0012	0.3761 ± 0.0144	0.9783 ± 0.0013	0.1121 ± 0.0037
weighted RF	0.5944 ± 0.0014	0.9336 ± 0.0014	0.5920 ± 0.0025	0.9953 ± 0.0001	0.4741 ± 0.0085	0.9991 ± 0.0001	0.7913 ± 0.0233

We also implement weighted random forest to build the prediction model and compare it with the conventional random forest-based model. The conventional random forest assigns equal weights to output labels (0,1) predicted by the decision trees of the random forest, while the weighted random forest assigns different weights to output labels (0,1), denoted as *w*_*i*_ = *n* _ *samples*/(2 ∗ *n*_*i*_), *i* = {0, 1}, where *n* _ *samples* denotes the number of samples in the training set, and *n*_*i*_ denotes the number of samples of each label (0,1). The performance of the weighted random forest is shown in Table 3. Compared with the conventional RF, the weighted RF produces better AUC but lower AUPR and F1. In general, the weighted RF and conventional RF have the similar performances in the miRNA-disease association prediction, and thus conventional RF classifier is finally adopted in this work.

### Comparison with existing state-of-the-art methods

To further demonstrate the advantages of NEMII, we compare it with three state-of-the-art methods: PBMDA [12], NTSMDA [11] and GRNMF [14], because they are latest methods with high-accuracy performances. PBMDA constructed a heterogeneous network based on miRNA-miRNA similarity, disease-disease similarity and known miRNA-disease associations, and then scored miRNA-disease associations by using the number of paths from miRNAs to diseases. NTSMDA considered topological information of the miRNA-disease association network, and used the network-based resource allocation algorithm. GRNMF integrated disease semantic similarity and miRNA functional similarity, and used a graph regularized nonnegative matrix factorization framework to predict associations. Here, we implement these prediction methods according to their publications, and evaluate all models by using five-fold cross-validation under same conditions.

As shown in Table 4, NEMII produces the best performances, achieving the AUPR score of 0.6104, and the AUC score of 0.9293. PBMDA, NTSMDA and GRNMF produce the AUPR score of 0.2095, 0.0916 and 0.2446, and the AUC score of 0.9164, 0.8857 and 0.9128 respectively. The AUPR score of NEMII is significantly higher than the other three methods, and the AUC score is also higher than the other three methods. Moreover, we analyze the statistical differences between NEMII and the other three methods in terms of AUC scores, and we observe that there exists a very significant difference between NEMII and other three methods: PBMDA (*p*-value = 0.001 by one-way ANOVA followed by post hoc Tukey test), NTSMDA (*p*-value = 0.001) and GRNMF (*p*-value = 0.001). Therefore, NEMII produces significantly better results than PBMDA, NTSMDA and GRNMF in the cross-validation experiment.

**Table 4:** Performances of NEMII, PBMDA, NTSMDA and GRNMF

Methods	AUPR	AUC	F1	ACC	REC	SPEC	PRE
NEMII	0.6104 ± 0.0012	0.9293 ± 0.0017	0.6147 ± 0.0025	0.9956 ± 0.0001	0.4893 ± 0.0060	0.9993 ± 0.0001	0.8289 ± 0.0164
PBMDA	0.2095 ± 0.0015	0.9164 ± 0.0005	0.2676 ± 0.0021	0.9892 ± 0.0005	0.2759 ± 0.0139	0.9944 ± 0.0006	0.2642 ± 0.0103
NTSMDA	0.0916 ± 0.0012	0.8857 ± 0.0009	0.1410 ± 0.0013	0.9740 ± 0.0015	0.2988 ± 0.0171	0.9788 ± 0.0017	0.0931 ± 0.0020
GRNMF	0.2446 ± 0.0024	0.9128 ± 0.0008	0.3192 ± 0.0137	0.9945 ± 0.0005	0.2989 ± 0.0127	0.9897 ± 0.0004	0.3066 ± 0.0016

**Fig. 2:**
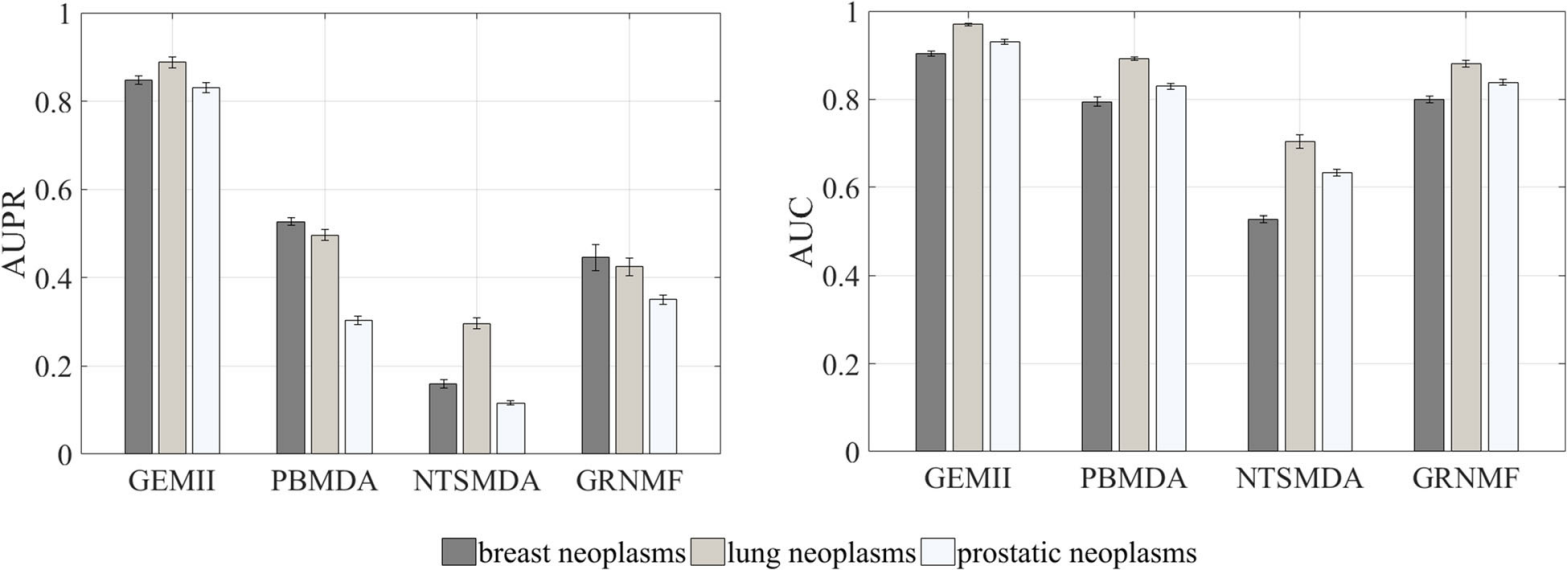
Performances of different methods on predicting miRNAs associated with three diseases

Further, we compare the predictive performances of four methods for specified diseases. We select three diseases of wide interests: “breast neoplasms”, “lung neoplasms” and “prostatic neoplasms”, and then we explore the results of different methods on these three diseases. Breast neoplasm develops from breast tissues which are highly prevalent in women. Lung neoplasm is a kind of malignant lung neoplasm caused by uncontrolled growth of lung tissue cells. Prostatic neoplasm is a kind of malignant neoplasm occurring in the prostate. We implement 10 runs of five-fold cross-validation and then obtain the prediction results of all these methods for each disease. As shown in Fig. 2 (left), NEMII produces significantly higher AUPR scores than PBMDA, NTSMDA and GRNMF for all three diseases. For example, the AUPR score for breast neoplasm produced by NEMII is 0.8476, which is 37.78% higher than the AUPR score of 0.5274 produced by PBMDA (the highest among PBMDA, NTSMDA, and GRNMF). And as shown in Fig. 2 (right), NEMII also produces higher AUC scores than PBMDA, NTSMDA and GRNMF for all three diseases. For example, the AUC score of prostatic neoplasm obtained by NEMII is 0.9296, which is 9.76% higher than 0.8389 obtained by GRNMF (the highest among PBMDA, NTSMDA, and GRNMF). Therefore, we can conclude that NEMII outperforms PBMDA, NTSMDA and GRNMF in predicting miRNAs for three specified diseases.

**Fig. 3:**
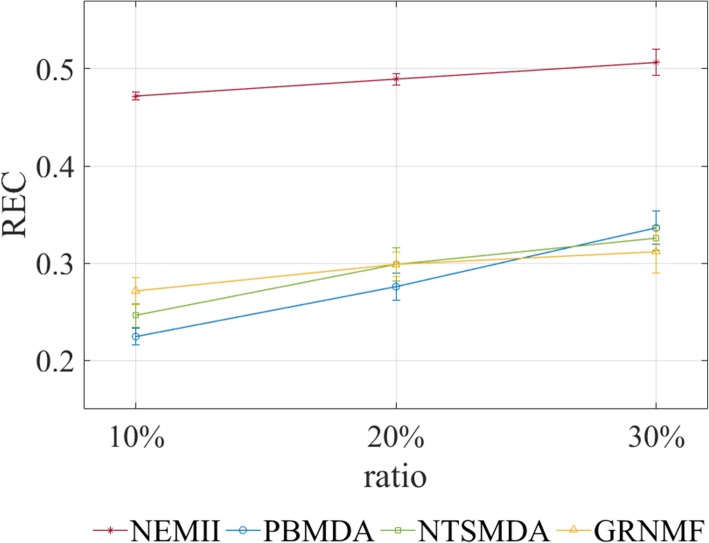
Performances of recovering associations of NEMII and other approaches

We further investigate what the percentage of removed known miRNA-disease associations could be covered by NEMII and other approaches. We randomly remove 10, 20 and 30% known miRNA-disease associations respectively, then train models based on the remained known and unknown associations and test what percentage of removed associations could be recovered. We use REC values to represent the results. The performances of NEMII and other approaches are shown in Fig. 3. Clearly, NEMII leads to better REC values than other approaches when removing 10, 20 and 30% known associations. To further demonstrate the advantage of NEMII, we conduct one-way ANOVA followed by post hoc Tukey test to test the difference between NEMII and other approaches in terms of REC values. The results show that NEMII produces significantly better results than PBMDA (*p*-value = 0.001), NTSMDA (*p*-value = 0.001) and GRNMF (*p*-value = 0.001). Therefore, we can conclude that NEMII outperforms PBMDA, NTSMDA and GRNMF in recovering the removed known miRNA-disease associations.

### Case studies

Here, we use case studies to test the capability of our method for predicting unknown miRNA-disease associations. We build the NEMII model by using all miRNA-disease associations in our dataset, and make predictions for non-association miRNA-disease pairs. Since all known associations in HMDD database are used to build models, the predicted associations have to be verified by public literature and other available sources. We list top 10 miRNA-disease associations predicted by NEMII in Table 5, and found evidence to confirm 5 out of them. For example, hsa-let-7c expression was found to be related to non-atrophic gastritis and atrophic gastritis [26]. Hsa-mir-103a-2 expression was downregulated in patients with myelodysplastic syndromes [27]. The expression of hsa-let-7e has an influence of time-dependent suppression on Biliary Atresia [28]. Increased expression of hsa-mir-1179 can inhibit breast cancer cell metastasis by modulating Notch signaling pathway [29]. The expression of hsa-mir-1179 was found to be associated with Hepatocellular Carcinoma. Therefore, our method can help to identify unknown miRNA-disease associations.

**Table 5:** The top 10 miRNA-disease associations predicted by our method

miRNA	Disease	Rank	Evidence
hsa-let-7c	Crohn Disease	1	N.A.
hsa-let-7c	Gastritis, Atrophic	2	[26]
hsa-let-7e	Lymphoproliferative Disorders	3	N.A.
hsa-let-7e	Giant Cell Tumors	4	N.A.
hsa-mir-103a-2	Myelodysplastic Syndromes	5	[27]
hsa-let-7e	Biliary Atresia	6	[28]
hsa-mir-10a	Carotid Artery Diseases	7	N.A.
hsa-mir-10b	Eczema	8	N.A.
hsa-mir-1179	Breast Neoplasms	9	[29]
hsa-mir-1179	Carcinoma, Hepatocellular	10	https://figshare.com/articles/Liver_hepatocellular_carcinoma/6804233

Moreover, we predict miRNAs which are associated with three diseases “breast neoplasms”, “lung neoplasms” and “prostatic neoplasms” mentioned in Comparison with existing state-of-the-art methods Section. Then we select the top 10 miRNAs associated with each disease, and try to obtain evidence to confirm our findings. As shown in Table 6, we have evidence to support 5 miRNAs associated with breast neoplasms. For example, miR-1179 expression was found to be frequently downregulated in breast cancer tissues and cell lines [29]. We find evidences to confirm that 5 miRNAs are associated with lung neoplasms. For example, hsa-mir-376c can suppress non-small-cell lung cancer cell growth and invasion by targeting LRH-1-mediated Wnt signaling pathway [35]. Moreover, 4 miRNAs are found to be associated with prostatic neoplasms. For example, hsa-mir-1179 was found to be one of the most highly upregulated miRNAs from the observation of micro dissected prostate tumor cells [39], and hsa-mir-10a was found to be one of the most highly expressed miRNAs in prostate tumors [39]. These evidence shows that these disease-related miRNAs have close relationships with breast neoplasms, lung neoplasms and prostatic neoplasms and may be of potential use in the diagnosis of these diseases. Therefore, NEMII is useful for predicting miRNAs associated with given diseases.

**Table 6:** Predicted miRNAs associated with three diseases

Disease	miRNA	Rank	Evidence
breast neoplasms	hsa-mir-1179	1	[29]
	hsa-mir-1180	2	[30]
	hsa-mir-106a	3	[31]
	hsa-mir-377	4	N.A.
	hsa-mir-1909	5	N.A.
	hsa-mir-181c	6	N.A.
	hsa-mir-1202	7	N.A.
	hsa-mir-1296	8	[32]
	hsa-mir-2110	9	N.A.
	hsa-mir-711	10	[33]
lung neoplasms	hsa-mir-1180	1	N.A.
	hsa-mir-1179	2	[34]
	hsa-mir-376c	3	[35]
	hsa-mir-500b	4	N.A.
	hsa-mir-1293	5	[36]
	hsa-mir-296	6	[37]
	hsa-mir-1183	7	N.A.
	hsa-mir-99b	8	[38]
	hsa-mir-298	9	N.A.
	hsa-mir-2110	10	N.A.
prostatic neoplasms	hsa-mir-103a-2	1	N.A.
	hsa-mir-1179	2	[39]
	hsa-mir-10b	3	N.A.
	hsa-mir-10a	4	[39]
	hsa-mir-1180	5	[40]
	hsa-mir-147a	6	N.A.
	hsa-mir-217	7	N.A.
	hsa-mir-125a	8	[41]
	hsa-mir-624	9	N.A.
	hsa-mir-630	10	N.A.

## Conclusion

The identification of miRNA-disease associations plays an important role in furthering understanding the molecular mechanism of many human diseases. In this work, we propose a novel computational method, called NEMII, to predict unknown miRNA-disease associations. Different from existing methods which mainly make use of biological features of miRNAs and diseases, NEMII extracts the embedding representations of miRNAs and diseases from the miRNA-disease bipartite network, and further combines them with biological features to build the prediction model. Experimental results reveal that NEMII performs better than the models using biological features alone and models using embedding representations alone, and SDNE produces better results than using other network embedding methods. NEMII also produces better results when compared with other state-of-the-art methods. Case studies show that NEMII can predict novel miRNA-disease associations, and can predict miRNAs associated with given diseases. In conclusion, NEMII is a promising method for the miRNA-disease association prediction.

## Methods

### Datasets

There are several databases about miRNA-disease associations, e.g. the human microRNA disease database (HMDD) [42], the database of differentially expressed miRNAs in human cancers (dbDEMC) [43] and the database for microRNA deregulation in human disease (miR2Disease) [44]. The databases lay the basis for developing computational methods to predict unobserved miRNA-disease associations.

In this study, we compile our datasets from HMDD database v2.0, miRBase and Medical Subject Heading (MeSH). HMDD [45] is a database which contains human miRNA-disease associations and comprehensive annotations. We downloaded experimentally confirmed miRNA-disease associations from HMDD, including 578 miRNAs, 383 diseases and 6448 associations. The database miRBase [46] is an online repository of miRNA sequences and the experimental miRNA-family relationships. We collected miRNA-family associations from miRBase, including 17,613 miRNAs and 1983 families; a miRNA belongs to a family and a family contains more than one miRNA. MeSH is a comprehensive medical vocabulary, which is useful for exploring the relationship between different diseases. We downloaded disease descriptors from MeSH. The relationships of diseases can be transformed into a directed acyclic graph (DAG), and the nodes of a DAG represent the diseases and the edges represent the relationships of different diseases. DAGs can be used to calculate disease semantic similarity [12].

We removed miRNAs without family information as well as diseases without MeSH descriptors. Finally, we obtained 4479 miRNA-disease associations between 412 miRNAs and 314 diseases, 278 miRNA-family associations between 412 miRNAs and 278 families, and MeSH descriptors for 314 diseases.

### Pipeline of network embedding-based multiple information integration method

For the following study, we first introduce several mathematical notations. Given miRNAs *M* = {*M*_1_, *M*_2_, …, *M*_*m*_}, diseases *D* = {*D*_1_, *D*_2_, ⋯, *D*_*n*_} and miRNA-disease associations, our task is to predict unknown miRNA-disease associations based on known associations and biological features. The associations between *m* miRNAs and *n* diseases can be represented by a binary matrix *A*, in which each row represents a miRNA and each column represents a disease. If the *i*th miRNA is associated with the *j*th disease, *A*_*ij*_ = 1; otherwise, *A*_*ij*_ = 0. Formally, *m* miRNAs, *n* diseases and their known associations can be formulated as a network, in which miRNAs and diseases are taken as nodes and their associations are taken as edges. The network can be represented by a (*m* + *n*) × (*m* + *n*) adjacency matrix *G*, defined as $$ G=\left[\begin{array}{cc}0& A\\ {}{A}^T& 0\end{array}\right] $$.

**Fig. 4:**
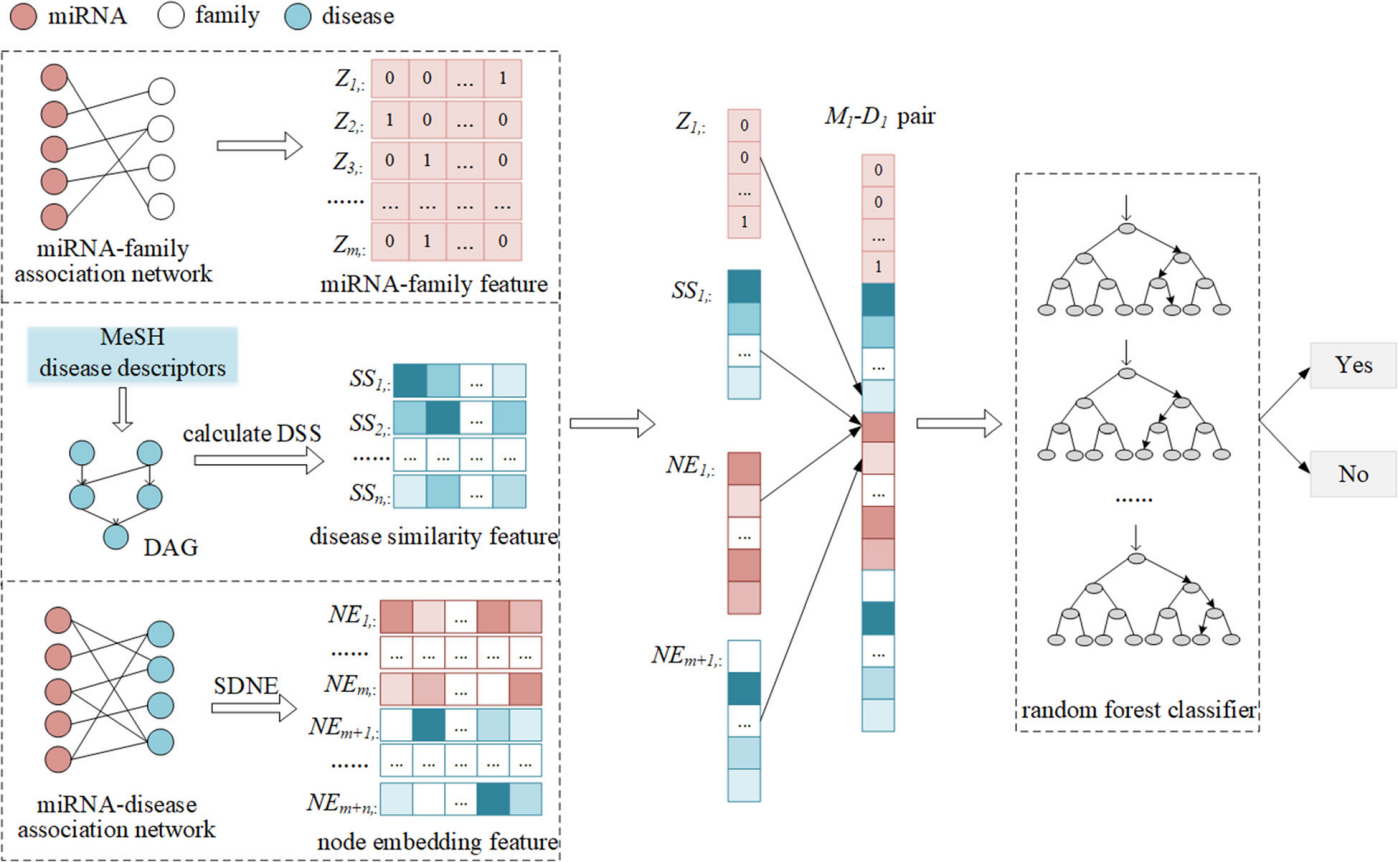
Pipeline of NEMII (DSS: Disease Semantic Similarity, SDNE: Structural Deep Network Embedding, DAG: Directed Acyclic Graph)

The studies [47–53] have revealed that combining diverse information helps to improve the accuracy of prediction models in bioinformatics. The network embedding-based multiple information integration method (NEMII) is to combine biological features of miRNAs and diseases with their embedding representations. As described in Fig. 4, NEMII takes several steps to construct a prediction model. First, miRNA-family associations are used to represent miRNAs; MeSH information of diseases are used to calculate disease-disease similarity and then represent diseases. Second, the known miRNA-disease associations are formulated as a bipartite network, and node embeddings in the bipartite network are learned by using SDNE and then used to represent miRNAs and diseases. Third, all representations of miRNAs and diseases are combined to represent miRNA-disease pairs. Finally, a prediction model is constructed based on the miRNA-disease pairs by using random forest.

### Constructing feature vectors to represent miRNA-disease pairs

In order to predict miRNA-disease associations, we should use a reasonable way to represent features of miRNA-disease pairs. To our best knowledge, most existing methods heavily rely on biological features of miRNAs and diseases, such as miRNA-family association, miRNA-functional similarity and disease semantic similarity. Besides, the features learned from the miRNA-disease association network can be taken into account. Features from known miRNA-disease bipartite network are seldom considered, but they are effective for preserving the property of the network. Therefore, there are three types of feature vectors for describing miRNA-disease pairs, i.e. vectors based on miRNA-family associations, vectors based on disease semantic similarity and vectors based on Structural Deep Network Embedding.

#### Representing miRNAs with miRNA-family associations

There is an assumption that miRNAs in the same family may perform similar biological functions. Here, we utilize miRNA-family associations to represent miRNA biological feature. For miRNAs *M* = {*M*_1_, *M*_2_, …, *M*_*m*_}, families *F* = {*F*_1_, *F*_2_, ⋯, *F*_*t*_}, and their associations, we can formulate them as a bipartite network, which uses miRNAs and families as nodes and uses their associations as edges. The bipartite network can be represented by a *m* × *t* adjacency matrix *Z*. This is, *Z*_*ij*_ = 1 if miRNA *M*_*i*_ belongs to family *F*_*j*_; otherwise, *Z*_*ij*_ = 0. Then, for a specific miRNA *M*_*i*_, we use the *i*th row vector of *Z,* namely *Z*_*i*, :_ to denote its biological feature.

#### Representing diseases with disease semantic similarity

Inspired by previous works [12], diseases and their relationships can be transformed into a directed acyclic graph (DAG), and DAGs can be used to calculate disease semantic similarity.

For a given disease *D*, the directed acyclic graph *DAG*_*D*_= (*V*_*D*_, *E*_*D*_). *V*_*D*_ denotes the node set including *D* and other diseases which have relationships with *D*, and *E*_*D*_ denotes the edge set which contains the links from parent disease to child disease. According to DAG, the semantic contribution of disease *d* in *DAG*_*D*_ to disease *D* can be denoted as:

$$ {S}_{\mathrm{D}}(d)=\left\{\begin{array}{c}1\  if\ d=D\\ {}\mathit{\max}\left\{{\Delta }_{\ast}\ast {S}_D\left({d}^{\prime}\right)|{d}^{\prime}\in children\ of\ d\right\}\  if\ d\ne D\end{array}\right. $$

Here, we set Δ_∗_ = 0.5. The semantic value of disease *D* can be calculated as:

$$ {SV}_D={\sum}_{d\in {V}_D}{S}_D(d) $$

The semantic similarity between disease *D*_*i*_ and disease *D*_*j*_ is calculated by:

$$ SS\left({D}_i,{D}_j\right)=\frac{\sum_{d\epsilon {V}_{D_i}\cap {V}_{D_j}}\left({S}_{D_i}(d)+{S}_{D_j}(d)\right)}{SV_{D_i}+{SV}_{D_j}} $$

where ${S}_{D_i}(d)$ is the semantic contribution of *d* to disease *D*_*i*_, and ${SV}_{D_i}$ is the semantic value of *D*_*i*_; for *D*_*j*_, the meanings of ${S}_{D_j}(d)$ and ${SV}_{D_j}$ are similar to *D*_*i*_. Then, the semantic similarity between all the diseases can be represented as a *n* × *n* matrix *SS*, and the value in row *i* and column *j* of *SS* represents the disease semantic similarity between *D*_*i*_ and *D*_*j*_. For a specific disease *D*_*i*_, we use the *i*th row vector of *SS*, namely *SS*_*i*, :_ to denote its biological feature.

#### Representing miRNA-disease pairs with structural deep network embedding

Recently, the network embedding methods show the great potentials of analyzing networks, especially extracting node features. Compared with traditional network analysis methods, which calculate network density, degree statistics and clustering coefficient, the network embedding methods generate low-dimensional vectors that reflect the comprehensive characteristic of networks. Since known miRNA-disease associations could be formulated as a miRNA-disease association network, we naturally use the network embedding methods to extract the features from it. We consider several popular network embedding methods in this work, and compare them in the Comparison with other network Embeddings and other classifiers Section. Because the Structural Deep Network Embedding method performs best among all embedding methods, it is finally adopted for the miRNA-disease association prediction.

Structural Deep Network Embedding method, namely SDNE, is semi-supervised deep model, which has multiple layers of non-linear functions to capture the highly non-linear network structure through first-order and second-order proximity. Since SDNE jointly optimizes first-order and second-order proximity, SDNE is robust to sparse networks [54], and outperforms popular network embedding methods in many applications, i.e. graph reconstruction, link prediction and visualization [55].

Given a network with *N* nodes and the adjacency matrix *G* = (*G*_*ij*_), we introduce how to learn the node embedding representations. SDNE utilizes the traditional deep autoencoder [56], which has two components: encoder and decoder. The encoder consists of multiple non-linear functions that maps initial representation of each node *x*_*i*_ to a low-rank space through *K* hidden layers, and the low-rank vector is denoted as *y*_*i*_. *x*_*i*_ = *G*_*i*, :_,where *G*_*i*, :_ is the *i* th row of the adjacency matrix *G* mentioned in Pipeline of network embedding-based multiple information integration method Section. The decoder attempts to reconstruct the representation of the node, and the reconstructed vector is denoted by ${\hat{x}}_i$.

The first-order proximity is used as the supervised information to preserve the local network structure, and its objective function is as follows:

1 $$ {L}_{1 st}={\sum}_{i,j=1}^N{G}_{ij}{\left\Vert \left({y}_i-{y}_j\right)\right\Vert}_2^2 $$

where *y*_*i*_ is the low-rank representation of node *i*, and *y*_*j*_ is the low-rank representation of node *j*.

The second-order proximity is used as the unsupervised information to capture the global network structure, and its objective function is as follows:

2 $$ {L}_{2 nd}={\left\Vert \left(\hat{X}-X\right)\odot B\right\Vert}_F^2 $$

where ⊙ means the Hadamard product. *B* is a *N* × *N* matrix. *B*_*ij*_ = 1, if *G*_*ij*_ = 0, else *b*_*ij*_ = *β*, where *β* is free parameter and *β* > 1. *X* = [*x*_1_, *x*_2_, ⋯, *x*_*N*_]^*T*^, $\hat{X}={\left[{\hat{x}}_1,{\hat{x}}_2,\cdots, {\hat{x}}_N\right]}^T$.

Moreover, a *L*2-norm regularization term is used to prevent overfitting and defined as follows:

3 $$ {L}_{reg}=\frac{1}{2}{\sum}_{k=1}^K\left({\left\Vert {W}^{(k)}\right\Vert}_F^2+{\left\Vert {\hat{W}}^{(k)}\right\Vert}_F^2\right) $$

where *K* is the number of hidden layers, *W*^*k*^ and ${\hat{W}}^{(k)}$ are the *k*th-layer weight matrices.

SDNE combines Eqs. 1, 2 and 3, and minimizes the following objective function:

4 $$ {\displaystyle \begin{array}{l}{L}_{mix}={L}_{2 nd}+\alpha {L}_{1 st}+\nu {L}_{reg}=\\ {}{\left\Vert \left(\hat{X}-X\right)\odot B\right\Vert}_F^2+\alpha {\sum}_{i,j=1}^N{G}_{ij}{\left\Vert \left({y}_i-{y}_j\right)\right\Vert}_2^2+\nu \frac{1}{2}{\sum}_{k=1}^K\left({\left\Vert {W}^{(k)}\right\Vert}_F^2+{\left\Vert {\hat{W}}^{(k)}\right\Vert}_F^2\right)\end{array}} $$

More details about SDNE are available in [54].

We can apply SDNE to the miRNA-disease bipartite network with the adjacency matrix *G*, and obtain a (*m* + *n*) × *d* embedding matrix *NE*, where *d* is a free parameter that denotes the dimension of node embeddings. *m* and *n* are mentioned in Pipeline of network embedding-based multiple information integration method Section. The rows of *NE*, namely *NE*_*i*, :_ correspond to the embeddings of *m* miRNA node and *n* disease nodes.

We also consider other popular network embedding methods Laplacian Eigenmaps (LE) [23], High-Order Proximity preserved Embedding (HOPE) [24] and DeepWalk [18], and compare SDNE with them in Comparison with other network Embeddings and other classifiers Section.

### Model construction

We combine three types of features to describe miRNA-disease pairs, and then use them to build classification-based models. Specifically, four feature vectors: miRNA-family feature vectors, disease semantic similarity feature vectors, miRNA embedding feature vectors and disease embedding feature vectors are merged. We adopt random forest as the classification engine to classify miRNA-disease pairs. Random forest is an ensemble learning method containing multiple classification trees [57]. Each tree is constructed by using a bootstrap sample of the training dataset. For each node within each tree, a randomly selected subset of the input features is used. Then the classification output of random forest is determined by the majority classification of all the trees. Random forest is well-known for its ability to deal with unbalanced datasets [58], and studies also demonstrated that random forest has good performances for bioinformatics problems [22, 59].

To the best of our knowledge, there are a great number of popular classifiers in bioinformatics, such as logistic regression, naive Bayes and support vector machine. We also compare random forest with these classifiers in Comparison with other network Embeddings and other classifiers Section.

## Additional file

Additional file 1: **Figure S1.** Five-fold cross-validation (CV) for Network Embedding-based Multiple Information Integration Method. (PDF 312 kb)

## Data Availability

The datasets generated and/or analysed during this study are available under open licenses in the data repository, https://github.com/BioMedicalBigDataMiningLab/NEMII.

## Abbreviations

AUC: Area under the receiver-operating characteristic curve
AUPR: Area under the precision-recall curve
DAG: Directed acyclic graph
dbDEMC: Database of differentially expressed miRNAs in human cancers
DSS: Disease semantic similarity
HMDD: Human microRNA disease database
HOPE: High-Order Proximity preserved Embedding
LE: Laplacian Eigenmaps
LR: Logistic regression
MeSH: Medical subject heading
miR2Disease: Database for microRNA deregulation in human disease
NB: Naive Bayes
NEMII: Network Embedding-based Multiple Information Integration method
RF: Random forest
SDNE: Structural Deep Network Embedding
SVD: Singular Value Decomposition
SVM: Support vector machine
