# Supplementary material for: A network embedding-based multiple information integration method for the MiRNA-disease association prediction
Source: BMC Bioinformatics. 2019 Sep 12;20:468. doi: 10.1186/s12859-019-3063-3 (PMC6740005; doi:10.1186/s12859-019-3063-3)
Supplement: Supplementary file 1 — Figure S1. Five-fold cross-validation (CV) for Network Embedding-based Multiple Information Integration Method. (PDF 312 kb) [file 12859_2019_3063_MOESM1_ESM.pdf]

Supplementary Material for A Network Embedding-based Multiple Information Integration Method for the MiRNA-disease Association Prediction

Yuchong Gong, Wen Zhang, Xiaohong Li

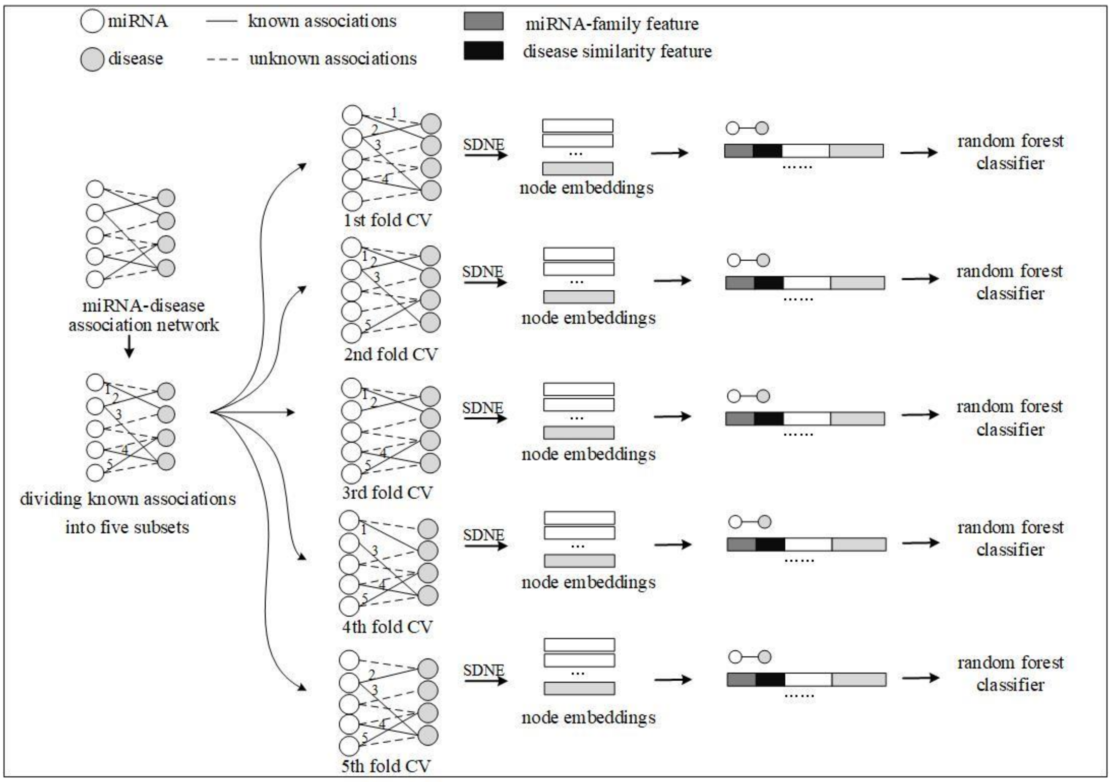


**Figure S1.** Five-fold cross-validation (CV) for Network Embedding-based Multiple Information Integration Method
